# Indigenous Sudanese sorghum‐based food: Secondary metabolites and antioxidant activities of traditional Sudanese nonalcoholic beverage *Hulu‐mur* from two sorghum landraces

**DOI:** 10.1002/fsn3.3275

**Published:** 2023-02-18

**Authors:** Tilal Sayed Abdelhalim, Aisha A. A. Abdalla, Khitma A. Sir Elkhatim, Mazahir H. Othman, Tagwa M. A. Mohammed Alkhair, Salah A. Almaiman, Magdi A. Osman, Amro B. Hassan

**Affiliations:** ^1^ Biotechnology and Biosafety Research Center Agricultural Research Corporation Shambat, Khartoum North Sudan; ^2^ Department of Food Science and Nutrition, College of Food and Agricultural Sciences King Saud University Riyadh Saudi Arabia; ^3^ Environment and Natural Resource and Desertification Research Institute (ENDRI) National Center for Research Khartoum Sudan

**Keywords:** antioxidant capacity, *Hulu‐mur*, sorghum‐based food, traditional

## Abstract

*Hulu‐mur* is a Sudanese traditional nonalcoholic beverage that is made from sorghum flour. This work determined the secondary metabolites and antioxidant activities of traditional Sudanese nonalcoholic beverage *Hulu‐mur* from two local sorghum landraces Abjaro and Hegarii. The changes on the total phenolic content (TPC), total flavonoid content (TFC), carotene content, tannins, and antioxidant activity (DPPH, reducing power, and FRAP) were estimated during the preparation of the *Hulu‐mur* flasks. For both landraces, a significant (*p* < .05) effect on the phytochemical compound and the antioxidant activity was observed during malting and fermentation of sorghum flour. However, the most increase in the TPC and carotene content was observed, whereas tannin and TFC were decreased in the *Hulu‐mur* flasks compared with the malted and fermented samples. The antioxidant activity DPPH, TRP, and FRAP was significantly (*p* < .05) higher in *Hulu‐mur* flasks than those of raw and processed flour. The partial least squares regression test stated a positive validation score of the *Hulu‐mur* flasks prepared from the both landraces. In conclusion, *Hulu‐mur* drink from Abjaro and Hegarii landraces contain high antioxidants compound, which could improve the health‐promoting metabolites in Sorghum‐based food.

## Graphical abstract

*Hulu‐mur* is a Sudanese traditional nonalcoholic beverage made from sorghum *Hulu‐mur* was prepared from sorghum landraces Abjaro and Hegarii. *Hulu‐mur* contains secondary metabolism with a higher antioxidant response. Production of *Hulu‐mur* could improve sorghum health‐promoting compounds.

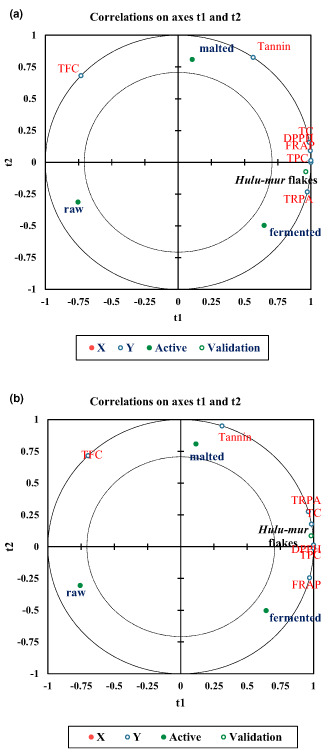

## 1 INTRODUCTION

Sorghum (*Sorghum bicolor* L. Moench) is one of the foremost vast cultivated carbohydrate‐rich crops of the Gramineae family and originated in Africa (Hossain et al., 2022). The sorghum crop is essential in semiarid regions of the world, mainly used for human consumption (de Morais Cardoso et al., 2017). For example, Sorghum is considered as the staple food for most of the people in Sudan (Abdelhalim et al., 2021). The highest proportion (80%) of the grain produced is consumed at the household level, with the remainder being used for sale and seed purposes. In addition, Dirar (1993) stated that sorghum grains are used to prepare different local sorghum‐based food such as leavened bread (*Kisra*), porridge (*Aceda*), thin fermented gruel (*Nasha*), alcoholic beverages (*Merissa*), and local nonalcoholic beverages (*Hulu‐mur*).

*Hulu‐mur* and *abreh* is a Sudanese traditional nonalcoholic beverage that is mainly consumed as a drink during the fasting of the holy lunar month of the Muslims, Ramadan (Dirar, 1993). In Arabic, the name *Hulu‐mur* means sweet–bitter, but the true meaning is sweet–sour, for the product is sour and not bitter. According to Dirar (1993), *Hulu‐mur*'s history dates almost 600 years ago, when the Muslim Arabs came to Sudan and married African women. These women face a Muslim husband who fasts during the holy Ramadan month and is quite hungry and thirsty in the evening. This stimulated the women to innovate such a unique beverage from brewing technology, based on traditional fermented foods, for breaking the fast after sunset (Agab, 1985).

In Sudan, the preparation of Hulu‐mur passes via unequaled rites and different domestic and traditional processing steps. The first step is the preparation of malted sorghum grains, which are locally named “Zurriea.” The grains are sprinkled with water from time to time for almost 4–5 days until the germination is completed (Dirar, 1993). This is the last phase of malting, which is locally given the name “Urrais” and is of fundamental importance in the fermentation process (Baidab et al., 2016). Secondly, the flour from the ungerminated grain is cooked instantly into a thick porridge similar to Aceda or slightly thinner medida. The malt flour is now added to the porridge while the latter is still hot. The two ingredients are thoroughly mixed with a wooden stirrer “Konosh.” During this process, liquefaction of the porridge occurs very rapidly so that within minutes, the mixture is already sweetish and fluid even though water has not been added to it. Consequently, the mixture is left to ferment and then the fermented dough is baked into thin brown sheets on a hot plate and then broken down into smaller flakes (Ibnouf, 2012). Lastly, for the preparation of the “Hulu‐Mur” drink, flakes are soaked in water and the supernatant is poured before serving (Agab, 1985).

According to Sulieman (2022), the major constituents of *Hulu‐mur* air‐dry flake are sugars (31%), protein (14.3%), lactic acid (3.8%), ash (3.5%), and starch (41%). Mariod et al. (2016) indicated that *Hulu‐mur* contains high levels of carbohydrates, protein, minerals (K, P, Fe, Mn), and essential amino acids (valine, isoleucine, leucine, tyrosine, and phenylalanine). Interestingly, the significant component that presumably goes into the solution is when the flakes are soaked in the sugar. Therefore, it is sensible to assume that Hulu‐mur has been contrived to provide a readily absorbable sugar, necessitating bridging the leeway in blood sugar caused by fasting (Agab, 1985; Dirar, 1993; Mahgoub et al., 1999).

However, so far, the changes on secondary metabolites and antioxidant activities in response to the *Hulu‐mur* production process have not been recognized yet. Therefore, this study aims to evaluate the changes in the phytochemical compounds and the antioxidant activity during the processing of traditional *Hulu‐mur* from two sorghum landraces Abjaro and Hegarii in Sudan.

## 2 MATERIALS AND METHODS

### 2.1 Plant materials

Two local landraces of Sudanese sorghum, namely Abjaro and Hegarii, were kindly obtained from the pioneer farmer at Fgwar Island, River Nile State in 2021. These landraces are taxonomically classified as durra type and predominantly cultivated as a winter crop. The grains were cleaned manually, freed from broken seeds and impurities, and stored in plastic bags until further use. Milling was carried out using a commercial laboratory mill. Whole grain flours were obtained and used to prepare raw (ungerminated) flour.

### 2.2 *Hulu‐mur* preparation

To prepare malt flour locally named “Zurriea,” approximately 2 kg of grains of each landrace were soaked in tap water overnight. Subsequently, the softened grains were spread on trays in a layer of roughly 4 cm and instantly covered with a wetted jute sack. The soaked grains were kept moist by frequent sprinkling with tap water for almost 4–5 days. After the germination occurred, and the plumule and radicles protruded out of the seeds coat, water spraying was stopped, and grains were kept dry for 2 days until their color turned reddish. Eventually, the sun‐dried reddish malt is then ground into a fine flour (malt flour) using a laboratory mill for further use in dough fermentation.

*Hulu‐mur* dough was prepared using the conventional Sudanese household method as previously described by Dirar (1993). Briefly, the flour from ungerminated grain was thoroughly mixed with distilled water in a ratio of 1:1.25 and after that cooked directly into a thick porridge. The hot porridge was then transferred from the cooking pot to another pot where fermentation occurred. The malt flour was then added to the hot porridge and thoroughly mixed with the wooden stirrer. Thenceforth, the whole mixture was further fermented for 36 h. at 35–37°C. The batter then became ready for flake preparation.

*Hulu‐mur*‐backed flakes were prepared from the fermented dough following the procedures of Dirar (1993). Hereto, the batter was thinned before the flakes baking by adding some water. Afterward, a suitable amount of the batter was poured on the hot plate “*Saj*.” Next, using a particular type of *gergeriba* (a piece of tough rubber or cardboard), the batter is spread toward the near side of the “*Saj*.” As the edge of the *Saj* is approached, the remainder of the batter carried by the *gergeriba* is piled back onto the baking sheet's surface. This process was repeated, spreading the extra batter backward and forward until all the batter aliquots were killed on the saj surface. Eventually, the *Hulu‐mur* flakes were ground and passed via a 0.4 mm screen for further analysis.

### 2.3 Preparation of extracts

The extracts of the samples were prepared in methanol at a ratio of 1:25 (w/v) as described by Talhaoui et al. (2014). The collected extracts were dried under vacuum using a rotary evaporator (Buchi Rotavapor® R‐300) and kept dry for further analysis.

### 2.4 Determination of total phenolic content

The total phenolic content (TPC) of the samples was determined spectrophotometrically following the Folin–Ciocalteu's reagent method (Waterhouse, 2002). The absorption of the phenolic solution was measured at 765 nm relative to a blank solution using a (UV)‐spectrophotometer (Elico SL150). Different concentrations of gallic acid dissolved in pure methanol were used to prepare the calibration curve (*R* ^2^ = .99743). The total phenolic content was then expressed as mg GAE/g DW.

### 2.5 Determination of total flavonoid content

The total flavonoid content (TFC) of the samples was measured according to the colorimetric assay (Kim et al., 2003). The absorbance of the solution was estimated at 510 nm. A calibration curve was prepared from the different concentrations of catechin (*R* ^2^ = .9761). The TFC was expressed as mg CE/g DW.

### 2.6 Determination of tannin content

The tannin content (TC) was estimated using the vanillin–HCl method as previously described by (Price et al., 1978). The absorbance of the solution was determined at 500 nm using an ultraviolet–visible spectrophotometer. Catechin concentrations were set to the standard curve, and tannin results were expressed as catechin equivalents (mg CE/g DW).

### 2.7 Determination of carotene content

The carotene content was extracted according to the method of Jacques et al. (2009). Approximately 2 g of powder of each *Hulu‐mur* ingredient was weighed and homogenized with 25 ml of cold acetone. The mixture was shaken for 10 min at room temperature before filtration. The supernatant was transferred to a separator funnel, where a liquid: liquid extraction was performed with 20 ml petroleum ether. The filtrate was washed with H_2_O, and the lower phase was discarded and then petroleum ether layer was filtered using Whatman No. 1 filter paper covered with 5 g of anhydrous sodium sulfate to remove residual water. The petroleum ether extracts were obtained, and the volume was adjusted to 25 ml with petroleum ether. The absorbance was measured at 450 nm, and the carotene content was expressed in μg/g DM.

### 2.8 Antioxidant activity assays

#### 2.8.1 DPPH scavenging activity

The DPPH scavenging activity of the *Hulu‐mur* ingredient extracts was measured spectrophotometrically according to Chang et al. (2001). The absorbance of the samples and the blank was determined at 517 nm using an ultraviolet–visible (UV–VIS PD‐303 UV) spectrophotometer. The DPPH scavenging was stated as Trolox equivalents per g (mg Trolox/g).

#### 2.8.2 Ferric reducing antioxidant power

The determination of Ferric Reducing Antioxidant Power (FRAP) of the extracts was made using U.V./visible spectrophotometer (593 nm) according to Benzie and Devaki (2017) method. FRAP results were expressed as micromoles of Trolox equivalents per g (mg Trolox/g).

#### 2.8.3 Total reducing power

The total reducing power (TRP) of the *Hulu‐mur* ingredient samples was determined by (Gulcin et al., 2002). Methanolic extracts were mixed with 2.5 ml of phosphate buffer (0.2 M, pH 6.6) and 2.5 ml of potassium ferricyanide (1%). The mixture was then incubated for 20 min at 50°C, and 2.5 ml trichloroacetic acid (10%) was added and centrifuged at 1038 *g* for 10 min. After that, 2.5 ml of the supernatant was mixed with 2.5 ml of distilled H_2_O and 0.5 ml of 0.1% ferric chloride. The absorbance of the mixture was measured at 700 nm. Ascorbic acid (AA) was used as a reference standard using the spectrophotometer, and results were expressed as AAE/g.

### 2.9 Statistical analysis

Data were checked for normality and homogeneity of variances using the Shapiro–Wilks and Levine's tests (*p* < .05). The main effects of Sudanese sorghum landraces, the *Hulu‐mur* production process, and their interactions were statistically analyzed by the two‐way analysis of variance (ANOVA) (GLM procedure in SAS) using the SAS 9.1 (SAS Institute) package for Windows. Treatments with significant differences were analyzed using Tukey's HSD post hoc test. The scatter plots between antioxidants assays and phytochemical compounds were developed using SigmaPlot software (version 14.5). Multivariate analysis was conducted using HJ‐Biplot PCA algorisms using XLSTAT software Version 2020.1.3 (Addinsoft) as described in Vidal et al. (2020). Linear partial least squares regression test (PLS) was performed to validate and optimize *Hulu‐mur* ingredients, using the XLSTAT software (Tenenhaus et al., 2005).

## 3 RESULTS

### 3.1 Phytochemical contents

The TPC, TFC, tannin, and total carotenoids (TC) of sorghum landraces were described in Table 1. There were significant (*p* < .05) TPC and TC variations among the sorghum landraces. While for total tannin and TFC, no noticeable varietal variation was encountered (Table 1). Regarding the *Hulu‐mur* preparation stages, a significant (*p* < .05) change was observed for almost all measured secondary metabolites. Except for total carotenoid, the interactions between the sorghum landraces and the *Hulu‐mur* production process remained significantly (*p* < .05) different (Table 1).

**TABLE 1:** Results of two‐factorial analysis of variance for secondary metabolites (TPC, TFC, tannin, and carotenoid contents) in grains of two sorghum landraces in response and processing types.

Cultivars	Phytochemicals compounds			
	Total tannin (mg CE/g DM)	TFC (mg CE/g DM)	TPC (mg GAE/g DM)	TC (μg/g DM)
Abjaro	7.4^a^	35.3^a^	73.0^a^	4.6^a^
Hejarii	7.1^a^	34.2^a^	71.2^b^	4.4^b^
*Hulu‐mur* processing steps				
Raw	2.2^d^	48.7^a^	43.9^d^	1.7^d^
Malted	12.3^a^	47.9^a^	69.8^c^	4.6^c^
Fermented	5.4^c^	17.3^c^	86.2^b^	5.5^b^
*Hulu‐mur* flakes	9.2^b^	25.0^b^	88.7^a^	6.1^a^
Two‐way ANOVA				
Cultivars, C	3.4^ns^	2.3^ns^	118.2**	11.7*
Processing, P	871.7***	502.7***	1672.2***	1112.3***
C × P	24.7***	6.1**	14.1**	0.7^ns^
SE±	0.81	2.9	3.7	0.35
CV%	5.0	5.0	1.5	3.2

*Note*: Data were evaluated via two‐way ANOVA, factors: two Sudanese sorghum landraces, and *Hulu‐mur* preparation stages. Identical letters indicate that values do not differ significantly at *p* < .05 according to Tukey HSD. Asterisks indicate significantly influential factors as follows: ns, not significant; * significant at *p* < .05; **Significant at *p* ≤ .01; ***Significant at *p* ≤ .001 level.

The highest tannin concentration was obtained in the malted flour, followed by *Hulu‐mur* flakes, and was significantly (*p* < .05) higher than the other *Hulu‐mur* preparation ingredients. However, the lowest tannin concentration was detected in the raw flour (Table 1). Contrary to tannin, the highest TFC was measured in the ungerminated flour followed by malted once and was significantly (*p* < .05) greater compared with the fermented dough and *Hulu‐mur* flakes (Table 1).

The total phenolic and carotenoid contents behaved similarly across *Hulu‐mur* preparation stages. *Hulu‐mur* flakes significantly possessed higher TPC and TC contents than the other *Hulu‐mur* ingredients. By contrast, the lowest TPC and TC were encountered in the ungerminated flour. The malted flour and fermented dough maintained significantly greater TPC and TC contents than ungerminated flour (Table 1).

### 3.2 In vitro antioxidant activity

The assays of DPPH scavenging activity (mg Trolox equivalent/g), TRP (mg ascorbic acid equivalent/g), and FRAP (mg Trolox equivalent/g) were used to measure the in vitro antioxidant activity in grains of Sorghum. The antioxidant activity in grains of Sorghum measured by three in vitro assays revealed a significant difference (*p* < .05) among sorghum landraces, processing and their possible interactions (Table 2).

**TABLE 2:** Results of two‐factorial analysis of variance for antioxidant activities (free radical scavenging DPPH [mg/g dry weight], total reducing power [AAE/g sample], and ferric reducing antioxidant power FRAP [mg/g dry weight]) of two Sudanese sorghum landraces in response to *Hulu‐mur* preparation stages.

Cultivars	In vitro antioxidant activity assays		
	DPPH (mg Trolox/g)	TRP (mg AAE/g)	FRAP (mg Trolox/g)
Abjaro	5.1^a^	4.5^a^	1.1
Hejarii	4.9^b^	4.5^a^	1.2
*Hulu‐mur* preparation stages			
Raw	3.2^d^	1.1^d^	0.6
Malted flour	5.0^c^	3.7^c^	1.0
Fermented dough	5.9^b^	5.3^b^	1.4^a^
*Hulu‐mur* flakes	6.2^a^	8.0^a^	1.7^a^
Two‐way ANOVA			
Cultivars, C	29.6***	0.1^ns^	4.3^ns^
Processing, P	2061.8***	3558.0***	149.8***
C × P	3.8**	94.5***	4.8**
SE±	0.25	0.53	0.1
CV%	1.5	2.6	8.6

*Note*: Data were evaluated via two‐way ANOVA, factors: two Sudanese sorghum landraces, and *Hulu‐mur* preparation stages. Identical letters indicate that values do not differ significantly at *p* < .05 according to Tukey HSD. Asterisks (*) indicate significantly influential factors as follows: ns, not significant; **Significant at *p* < .01; ***Significant at *p* < .001 level.

Significant total antioxidant activity changes were in response to *Hulu‐mur* preparation stages (Table 2). Detectable varietal variations were noticed for the free radical scavenging DPPH assay. The interactions between sorghum landraces and the *Hulu‐mur* production process stayed significantly different (Table 2).

*Hulu‐mur* flakes maintained the highest DPPH, TRP, and FRAP contents and were significantly different from the other *Hulu‐mur* ingredients. At the same time, the lowest antioxidant activity was encountered in the ungerminated flour. There were no significant (*p* > .05) in FRAP contents between the *Hulu‐mur* flakes and fermented dough (Table 2).

### 3.3 Relationship between the measured phytochemicals and in vitro antioxidant activity

**FIGURE 1:**
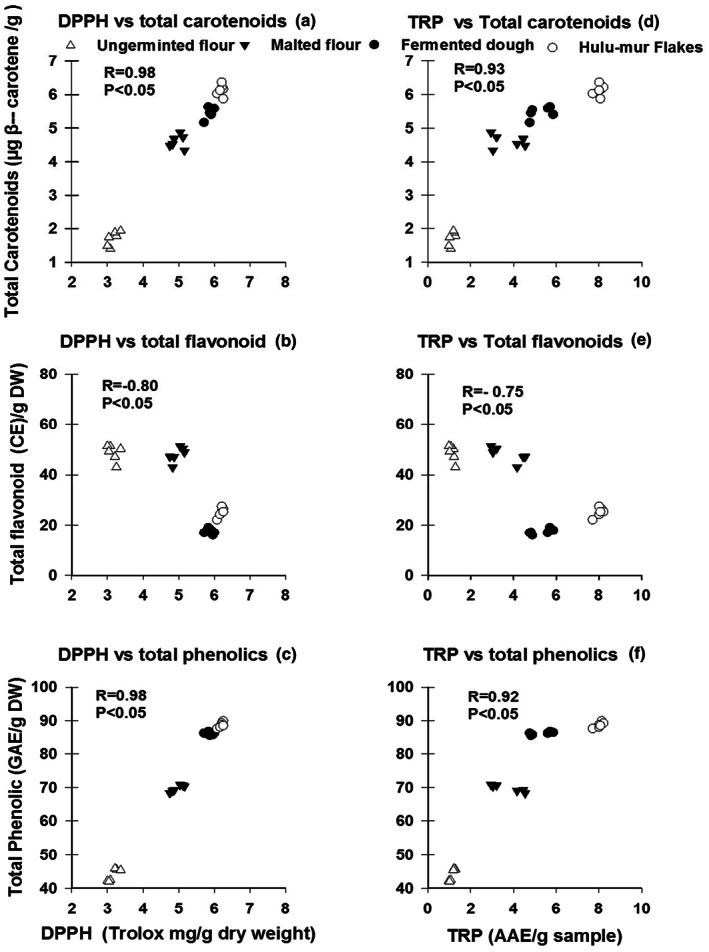
Scatter plots of free radical scavenging DPPH versus total carotenoids (a), total flavonoids (b) and total phenolic (c), and between total reducing power (TRP) and total carotenoid (d), total flavonoids, (e) and total phenolic (f).

**FIGURE 2:**
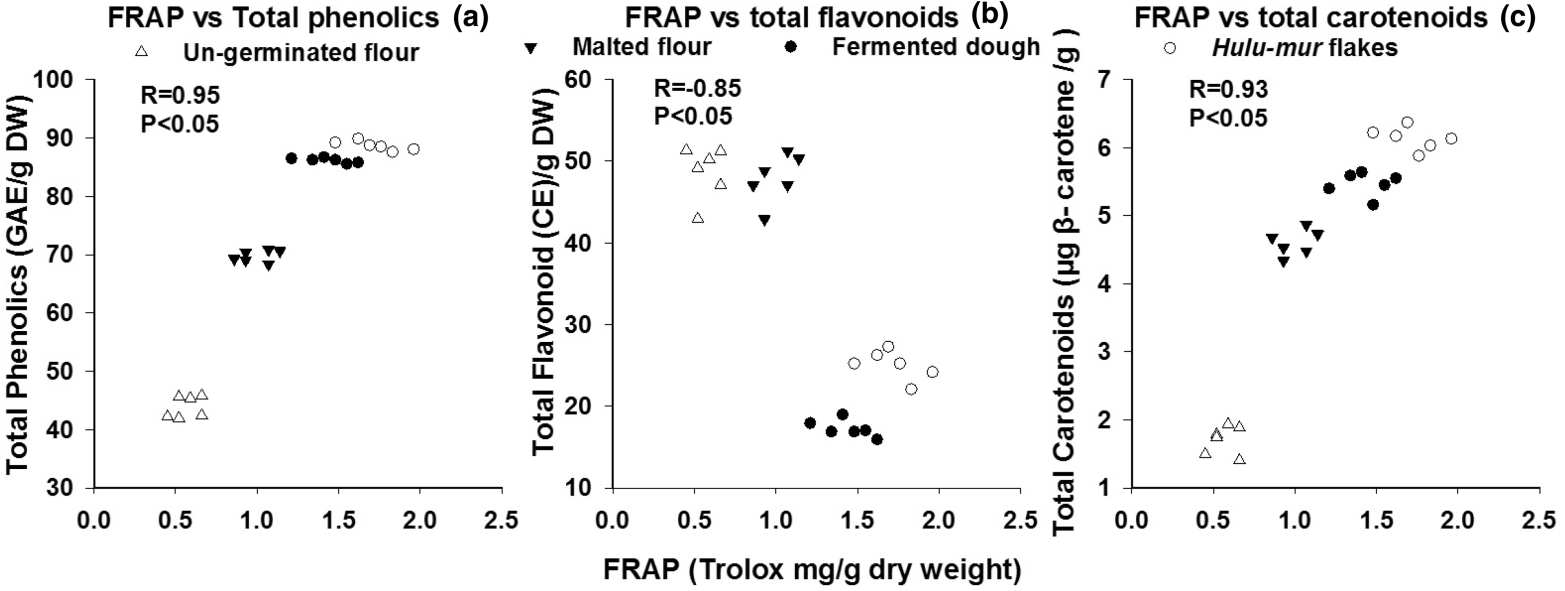
Scatter plots of ferric reducing antioxidant power FRAP versus total phenolic content (a); total flavonoid content (b), and total carotenoid (c).

A significant positive correlation was detected between DPPH values and TC content (*r* = .98; *p* < .05) (Figure 1a), and between DPPH values and TPC (*r* = .98; *p* < .05) (Figure 1c), while there was an inverse relationship between the DPPH values and TFC (*r* = −.80; *p* < .05) (Figure 1b) Also, TRP was reported to be positively correlated with total carotenoids (*r* = .93; *p* < .05) (Figure 1d) and between TRP and total phenolic contents (*r* = .98; *p* < .05) (Figure 1f). Once again, an inverse relationship between TRP and total flavonoid was reported (*r* = −.75; *p* < .05) (Figure 1e) A similar trend was observed for FRAP values, as there were positive relationships between FRAP values and TPC (*r* = .95; *p* < .05) (Figure 2a) and between FRAP values and TC (*r* = .93; *p* < .05) (Figure 2c), whereas there was a negative correlation between FRAP values and TFC (*r* = −.85; *p* < .05) (Figure 2c).

### 3.4 Multivariate analysis

**FIGURE 3:**
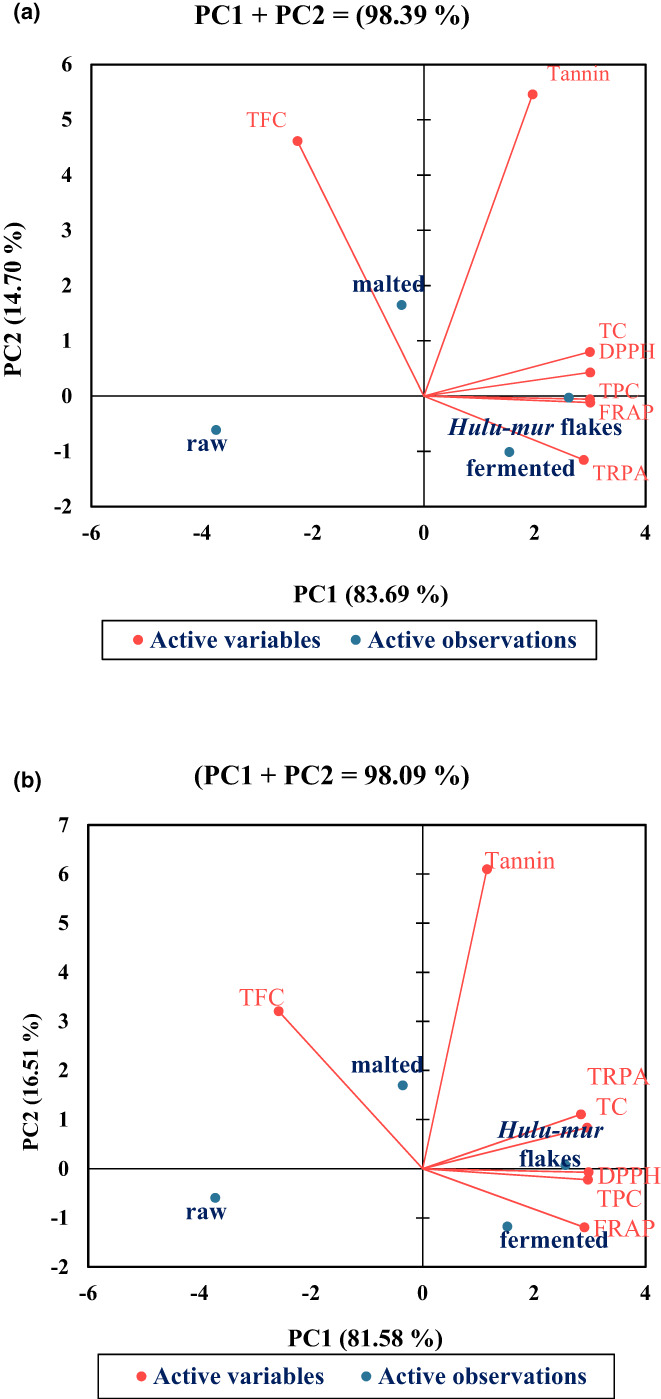
Principal component of analysis (PCA) scores for the experimental variables determined in grains of two Sudanese sorghum landraces, Abjaro (a) & Hejarii (b), in response to *Hulu‐mur* preparation stages.

The principle component of analysis (PCA) biplot revealed an apparent clustering of the *Hulu‐mur* preparation stages ingredients from the two sorghum landraces Abjaro and Hegarii (Figure 3a,b). For the Abjaro sorghum landrace, the scree plot indicates that the first two principal components account for 98.39% of the total variance (Figure 3a). The component PC1 explained 83.7%, and PC2 explained 14.7% of the total variation (Figure 3a). While for the Hegarii landrace, the axes contribution of the PC1 and PC2 present 81.58% and 16.51% resulting in high variability of 98.09% (Figure 3b). The PCA factor loading showed that TPC, tannin, total carotenoids, DPPH, TRP, and FRAP were greatly associated for both landraces with PC1. By contrast, TFC was highly correlated with PC2 (Figure 3a,b). Accordingly, the PCA factor loading shows a strong positive correlation between the TPC, TC, and antioxidant activities with the fermented dough and *Hulu‐mur* flakes.

**FIGURE 4:**
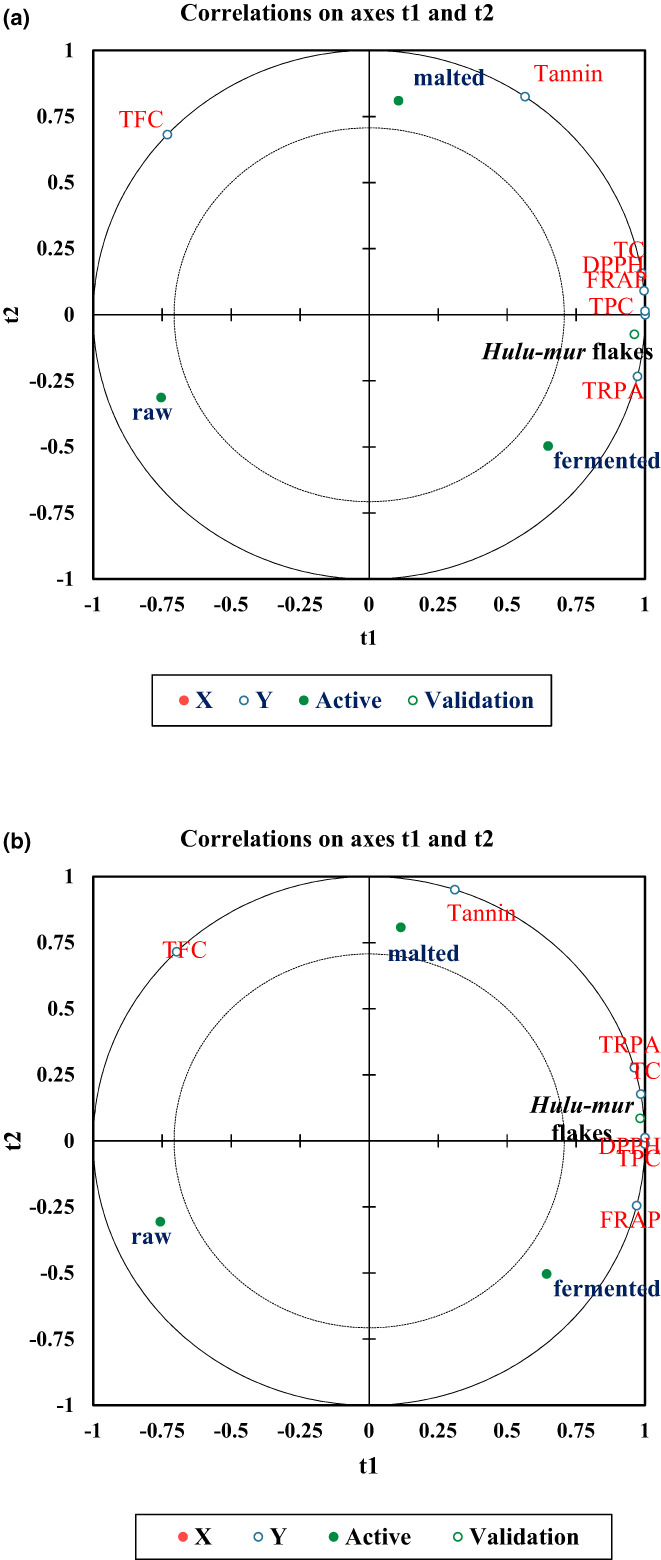
Partial least squares regression analysis (PLS) for the experimental variables determined in grains of two Sudanese sorghum landraces, Abjaro (a) & Hejarii (b), across different *Hulu‐mur* preparation stages.

Partial least squares analysis described the interactive impacts of *Hulu‐mur* preparation stages ingredients (*x* variables) on the stated factors (*y* variables) of the two sorghum grain landraces Abjaro (Figure 4a) and Hejarii (Figure 4b). Referring to the PLS model, the fermented dough and *Hulu‐mur* flakes showed a positive validation score for TPC, TC, DPPH, TRPA, and FRAB. However, PLS specified that the *Hulu‐mur* flakes of Abjaro and Hejarii's landraces were reflected as the most valid one.

## 4 DISCUSSION

The traditional nonalcoholic beverage “*Hulu‐mur*” is commonly prepared from the Sudanese sorghum variety, Feterita, which provides the final product with its distinguished red color (Agab, 1985). Despite several previous investigations evaluating the nutritional value of the *Hulu‐mur*, however, most of these studies aimed to measure the chemical composition, minerals, sugars, and vitamins (Baidab et al., 2016; Ibnouf, 2012; Mahgoub et al., 1999; Mariod et al., 2016; Taylor & Duodu, 2019). So far, evaluation of the changes in the phytochemical compounds and the antioxidant activity during the processing of this Sudanese traditional nonalcoholic beverage, particularly from other sorghum genotypes rather than Feterita, has not been investigated yet. In this regard, in this study, we targeted to examine the production of *Hulu‐mur* from different types of sorghum landraces, Abjaro and Hegarii landraces classified as durra types.

Generally, the obtained results revealed that both Abjaro and Hegarii landraces contain high amounts of phenolic compounds with high antioxidant activity. During *Hulu‐mur* processing from both landraces, significant positive changes in TPC, tannin, TC, and antioxidant activity (DPPH, TRPA, and FRAB) were observed. However, the higher values were obtained from both landraces at the final products, Hulu‐mur flask. These changes might be associated with the traditional processing related to the *Hulu‐mur* such as germination, malting, fermentation, and hot‐plate cooking.

The germination process of grains might lead to activating enzymes that are not active in raw seeds, resulting in structural modification and the construction of new compounds, thus improving grains' palatability and nutritional value (Budhwar et al., 2020). Additionally, an increase in total phenolic compounds and DPPH inhibition activity after sorghum germination was described by Singh et al. (2019). Moreover, Hithamani and Srinivasan (2014) observed an increase in the content of total soluble phenols, tannins, and phenolic acids upon sorghum germination. Furthermore, Donkor et al. (2012) observed an enhancement in phenolic level following sorghum germination.

Fermentation is a vital process brought by microorganisms and their enzymes which cause a biochemical modification of the primary food matrix (Kahajdova & Karovicova, 2007). Fermentation is extensively used to produce traditional food at the household level in low‐income countries. Concerning fermentation is a proven sustainable approach, which enhances the bio‐accessibility and bioavailability of nutrients from different fermented food (Zhang et al., 2012). The effect of fermentation on sorghum phytochemicals has been examined in many studies. For example, Zaroug et al. (2014) reported that fermentation of nontannin Sorghum for 8 and 24 h. showed an increase in the total phenol and flavonoid content and antioxidant capacity compared with raw Sorghum. Moreover, Wang et al. (2014) stated that microbial fermentation caused an incremental increase in certain cereals' phenolic compound and antioxidant activity.

The detected enhancement of the phenolic compounds and their antioxidant activity of the malt sorghum flour could be due to the increased activity of induced endogenous enzymes, which might release the bound or conjugated phenolic compounds. Accordingly, the malting process was necessary to engorge the endogenous antioxidant activity and increase phenolic compound development. Lu et al. (2007) observed positive changes in the TPC, TFC, and antioxidant activity during the malting of barley grains.

During cooking, hot‐plate cooking in this study, the breakdown of rigid cell walls might be a reason for increasing the total phenol content and its extractability (Adefegha & Oboh, 2011). Dewanto et al. (2002) observed an extreme increment of the phenolic in corn, wheat, and oats cell walls after cooking for 10–50 min.

Observations in the PCA (Figure 3a,b) declared that fermented dough and *Hulu‐mur* flakes improved the accumulation of health‐promoting phytochemicals and antioxidant activity of Sorghum, whereas malted flour improved only the TFC for both sorghum landraces. Furthermore, the step of *Hulu‐mur* flakes of Abjaro and Hejarii's landraces (Figure 4a,b) show the most valid processing step among other steps. Hence, validation test revealed that the production of *Hulu‐mur*, the Sudanese traditional nonalcoholic beverage from these landraces, might significantly improve the health‐promoting metabolites of Sorghum‐based food.

In general, based on the obtained results, *Hulu‐mur* flasks prepared from both Abjaro and Hegarii landraces exhibited high content of the secondary metabolites and antioxidant activities. Therefore, they might be considered functional sorghum‐based foods and associated with substantial health‐promoting metabolites that contribute to the health benefits. On the contrary, the making of *Hulu‐mur* as a local nonalcoholic beverage from different Sudanese durra types, rather than Sorghum variety Feterita, should let the industry to produce traditional sorghum‐based foods and drinks rich with secondary metabolism and antioxidants.

## 5 CONCLUSION

The traditional process of producing *Hulu‐mur* flask, the Sudanese traditional nonalcoholic beverage, differently affected the contents of Secondary metabolites and antioxidant activity values. The phytochemical compound and the antioxidant capacity were enhanced during malting and fermentation of sorghum flour. However, the highest values were found significantly higher in *Hulu‐mur* flask obtained from Abjaro and Hegarii landraces. Moreover, regarding the PLS, the final *Hulu‐mur* product from both landraces is revealed as the greatest valid, which represents an excellent way to increase the secondary metabolites and antioxidant activities. Hence, *Hulu‐mur* initially could be considered for the food industry as traditional sorghum‐based foods and drinks rich in some secondary metabolism and antioxidants.

## CONFLICT OF INTEREST STATEMENT

The authors declare that there are no conflicts of interest.

## ETHICS STATEMENT

Ethics approval was not required for this research.

## Data Availability

Research data are not shared.
